# Understanding the Barriers and Facilitators to Sharing Patient-Generated Health Data Using Digital Technology for People Living With Long-Term Health Conditions: A Narrative Review

**DOI:** 10.3389/fpubh.2021.641424

**Published:** 2021-11-23

**Authors:** Emma Simpson, Richard Brown, Elizabeth Sillence, Lynne Coventry, Karen Lloyd, Jo Gibbs, Shema Tariq, Abigail C. Durrant

**Affiliations:** ^1^The NHS Business Services Authority, Newcastle upon Tyne, United Kingdom; ^2^Department of Psychology, Northumbria University Newcastle, Newcastle upon Tyne, United Kingdom; ^3^Institute for Global Health, University College London, London, United Kingdom; ^4^Open Lab, School of Computing, Newcastle University, Newcastle upon Tyne, United Kingdom

**Keywords:** data sharing, patient-generated health data, technology, long-term health conditions, trust, identity, privacy, security

## Abstract

Using digital technology to share patient-generated health data has the potential to improve the self-management of multiple long-term health conditions. Sharing these data can allow patients to receive additional support from healthcare professionals and peer communities, as well as enhance their understanding of their own health. A deeper understanding of the concerns raised by those living with long-term health conditions when considering whether to share health data *via* digital technology may help to facilitate effective data sharing practices in the future. The aim of this review is to identify whether trust, identity, privacy and security concerns present barriers to the successful sharing of patient-generated data using digital technology by those living with long-term health conditions. We also address the impact of stigma on concerns surrounding sharing health data with others. Searches of CINAHL, PsychInfo and Web of Knowledge were conducted in December 2019 and again in October 2020 producing 2,581 results. An iterative review process resulted in a final dataset of 23 peer-reviewed articles. A thorough analysis of the selected articles found that issues surrounding trust, identity, privacy and security clearly present barriers to the sharing of patient-generated data across multiple sharing contexts. The presence of enacted stigma also acts as a barrier to sharing across multiple settings. We found that the majority of literature focuses on clinical settings with relatively little attention being given to sharing with third parties. Finally, we suggest the need for more solution-based research to overcome the discussed barriers to sharing.

## Introduction

Over the last several decades there has been a substantial increase in life expectancy across the industrialized world due to advancements in digital technology and medicine, as well as successful public health initiatives (1, 2). Despite this achievement, an aging society has come with a rise in the prevalence of long-term health conditions (LTHCs) (3). Many LTHCs are supported by continuous self-monitoring and management. Advancements in digital technology have provided the opportunity for people to collect, manage and share personal health data to better manage their own health and achieve better health outcomes and quality of life. People living with LTHCs often record, monitor and manage personal health data, which encompasses a broad range of personal health information such as medication adherence, health and lifestyle practices and experiences of health, that patients may choose to share with others. These patient-generated health data (PGData) have the potential to improve the self-management of multiple conditions and, when shared with healthcare providers, improve the provision of care (4, 5).

There are multiple benefits to sharing PGData. Sharing these data can lead to a feeling of increased support when interacting with peer communities (others living with the same or similar condition), family or friends, as well as leading to better healthcare decision making in patients (6, 7). Using PGData from electronic devices has been shown to improve patient outcomes in a range of conditions such as diabetes, obesity, heart disease, and other chronic conditions (8). For example, in a study of cancer patients, the use of a digital app on an electronic tablet helped to improve patients' recall of symptoms and enabled the sharing of health information with clinicians (9). Cancer patients have also been reported to be willing to share PGData with cancer registries where they recognize the benefits for personal health management and population health (10). Patients who share PGData *via* digital platforms such as PatientsLikeMe report the greatest benefits to sharing as being able to learn more about their symptoms and to understand the side effects of their treatment (11). Furthermore, the increased sharing of PGData with third parties may allow big data public health practices to identify previously concealed patterns among the reported experiences of multiple LTHCs, which may help to optimize the delivery of care for individual patients (12, 13). Ultimately, the use of PGData in the management of health conditions enhances understanding and generates a holistic picture of one's personal health and disease management (14, 15).

There are a number of factors that facilitate the sharing of PGData, such as individual altruistic tendencies and the seeking of social support (16). Conversely, factors that are considered barriers to the sharing of PGData include poor health literacy and the perceived burden of having to manage data associated with one's condition(s) (17). The growing prevalence of digital technology in the transmission of personal health data would suggest that issues surrounding Trust, Identity, Privacy and Security (TIPS) are likely to be an increasing and evolving concern. For example, TIPS concerns have been found to be critical when seeking to facilitate the sharing of PGData among those living with HIV (18). This narrative review is conducted as part of a UK EPSRC funded programme (“INTUIT: Interaction Design for Trusted Sharing of Personal Health Data to Live Well with HIV”, 2020) (19) examining TIPS concerns around the sharing of PGData primarily among those living with HIV, but also looks to investigate TIPS concerns among those living with a range of other LTHCs. The INTUIT project aims to contribute toward removing barriers to collecting and sharing PGData in order to improve the health and well-being of stigmatized populations. The sharing of PGData raises multiple TIPS concerns for those living with LTHCs and may hold particular significance for those with potentially stigmatized conditions due to fears of discrimination or other harmful consequences. People who anticipate experiences of stigma as a result of their LTHC(s) are likely to be more guarded when reporting their experiences of health, which may prevent them from receiving an appropriate level of care (20, 21). Therefore, understanding the role that both stigma and TIPS concerns play in the sharing of PGData with others, by those living with LTHCs, may help to promote effective data-sharing practices, potentially leading to improved delivery and self-management of care.

The potential benefits of PGData for understanding a range of health conditions and for optimizing delivery of care may help to support the rising prevalence of LTHCs. The use of PGData has the potential to transform the delivery of healthcare and to improve the management of countless LTHCs (4). However, cultivating an ecosystem that protects the interests of patients and builds confidence that healthcare systems will use personal information responsibly presents unique challenges to researchers, designers and policy makers working in digital health. To realize the benefits of PGData we must first understand the barriers and facilitators to sharing using digital technology for people living with LTHCs. To address this, we have conducted a narrative review of previous literature addressing TIPS concerns and the role of stigma in the sharing of PGData *via* digital technology by those living with LTHCs. The research questions directing this narrative review are (i) do TIPS concerns present a barrier to the successful sharing of PGData using digital technology by people living with LTHCs; and (ii) what is the impact of stigma on the sharing of PGData *via* digital technology by those living with LTHCs? By addressing these research questions, we aim to discuss barriers and facilitators to the effective sharing of PGData across multiple contexts: sharing with clinical staff, public health surveillance, researchers, peer communities, friends, social networks and other third-party organizations.

## Methods

### Narrative Review

Narrative reviews are fast becoming the most common form of literature review across multiple disciplines (22). Though the literature is summarized in a way that is not explicitly systematic, narrative reviews nevertheless provide a comprehensive synthesis of up-to-date evidence for researchers, designers and policy makers working in the field of digital health (22–24). The synthesis of qualitative and quantitative research is critical to ensuring that patient experiences, needs and preferences are understood and taken into consideration when designing and implementing healthcare technology (24). In conducting this narrative review, a scale for the quality assessment of narrative review articles (SANRA) was consulted in order to ensure that it meets the expected standards for this category of review (22). This narrative review aimed to better understand issues of Trust, Identity, Privacy and Security (TIPS) in those living with LTHCs when using digital technology to share their personal health and lifestyle data. This review also explores the role that stigma plays in sharing this data *via* technology by people with LTHCs.

### Inclusion and Exclusion Criteria

This narrative review was conducted by first establishing the inclusion and exclusion criteria for article selection, which was agreed by the whole research team (see Table 1). The LTHCs featured in this inclusion criteria were in line with the wider goals of the INTUIT project and based on the findings of previous research that discussed experiences of stigma among those living with HIV (18, 25, 26), other sexually transmitted infections (27, 28), diabetes (29–31) and Mental Health conditions (32–34). Our inclusion criteria also sought to capture those LTHCs considered most prevalent and impactful on society (cancer, cardiovascular disease and dementia) (35).

**Table 1:** Inclusion and exclusion criteria for selecting peer-reviewed articles.

**Inclusion criteria**	**Exclusion criteria**
• Addresses any of the selected LTHCs (HIV, diabetes (types 1, 2 and unspecified), mental health, sexual health, cancer, cardiovascular disease or dementia); and • Includes a type of communication (with peers, with clinical staff or with public health surveillance); and • Includes a form of digital technology (social media, online forums, mobile apps or other digital platforms); and • Addresses the sharing of PGData; and • A barrier to sharing; or • A facilitator to sharing; or • Considers issues surrounding Trust, Identity, Privacy and Security.	• Addresses the sharing of generic health promotion/education/ information; or • Focusses on a specific LTHC outside of the selected categories; or • Does not present empirical data.

### The Initial Search

The inclusion and exclusion criteria were then applied to an initial search exercise conducted in December 2019. This initial search was conducted by one member of the research team and involved a search of the available published literature using the following databases: CINAHL, PsychInfo, Web of Knowledge and by referring to the reference lists of relevant articles. An iterative searching strategy was developed as the language and terminology pertaining to PGData became more familiar to the researcher. Within current health literature, there are multiple variations of terms that are used to describe PGData, including “personal health information,” “personal health data,” “patient-authored information,” “patient-generated information,” “protected health information,” whereas other literature may simply refer to the data as “medical information”. Combinations of words and strings representing the sharing of PGData were applied to the selected databases with Boolean operators “AND” and “OR” to broaden the search. This initial search exercise yielded 2,479 results.

### Refining the Search

One member of the research team collected the initial articles from the various sources. Duplicates were removed. An iterative process of reading the titles and excluding search results whose titles indicated that they did not satisfy any of the inclusion criteria or contained a relevant feature of the exclusion criteria (see Table 1). The abstracts and texts of search results whose titles passed this initial inspection were then reviewed by three members of the research team to determine their relevancy in accordance with the full inclusion and exclusion criteria, thus progressively refining the scope of the initial search.

### Article Selection

Three members of the research team independently reviewed the list of potentially relevant articles against the inclusion and exclusion criteria. A meeting was held to compare lists and agree which to take forward. Any articles where one member of the team had identified them for inclusion were discussed and a decision made by mutual agreement. One member of the research team meticulously reviewed the full text for articles that the research team identified as potentially (though not certainly) relevant to the directives of the review. For example, for articles that addressed various health conditions, the researcher examined the text to ensure that significant attention was given by the candidate article to the sharing of personal health information associated with LTHCs. This member also extracted any relevant articles from the references of the candidate articles. Each time new articles were identified the three first reviewers would meet and discuss their inclusion. The full research team evaluated and discussed the short list of candidate articles with respect to the selection criteria and were given the opportunity to suggest any articles known to them that had been missed. This process resulted in 19 peer-reviewed articles being selected by mutual agreement.

### Updating the Search

The search, refinement and selection processes described above were repeated in October 2020 to identify further contributions that had been made to the literature since the initial search. The second search produced a further 102 results, four of which were selected for inclusion in the narrative review.

### Review

The final dataset comprised 23 peer-reviewed articles. The results from the articles were extracted into Microsoft Excel before NVivo 12 was used to thematically analyse the data. The thematic analysis of the selected articles was undertaken by all members of the research team and involved an iterative review of the findings in consideration of their relevance to the two research questions stated above. All members of the research team mutually discussed the results of the selected articles and subsequent thematic analysis in order to synthesize and present the findings below.

## Findings

The review of the selected articles finds that issues surrounding Trust, Identity, Privacy and Security clearly present barriers (but in some cases facilitators) to the sharing of PGData across all contexts (i.e., sharing with clinical staff, public health surveillance, researchers, peer communities, friends, social networks and other third-party organizations). Examples of the specific TIPS issues referred to in the literature, along with a brief overview of the selected articles, are presented and discussed below to provide a review of the literature thus far. Table 2 provides a description of all of the articles included in this review.

**Table 2:** Included papers overview.

**References**	**Country of origin**	**Aim/purpose**	**Long-term health condition (LTHC)**	**Population**	**Sharing data with/platform**	**Key findings**
Agaku et al. (36)	USA	This study assessed the perceptions and behaviors of US adults regarding the security of their protected health information (PHI).	Various conditions	*n* = 1,452 adults	Healthcare professionals (HCPs)	This study reported that most US adults are concerned about the security and privacy of their PHI, and that such concerns are associated with an increased likelihood of non-disclosure of sensitive information to HCPs.
Ancker et al. (17)	USA	This study investigated how patients with multiple chronic conditions (MCC) manage their personal health records and information sharing with HCPs. This study also addressed how patients perceive their own role in managing their health information.	MCC	*n* = 22 adults	HCPs	Personal health information management should be recognized as an additional burden that MCC places upon patients. Effective structural solutions for information sharing, whether institutional ones such as care management or technological ones such as electronic health information exchange, are likely not only to improve the quality of information shared but reduce the burden on patients already weighed down by MCC.
Bernaerdt et al. (37)	Belgium	This study investigated the perceptions and attitudes of vulnerable patients regarding sharing medical information with HCPs and third parties *via* a digital platform.	Various conditions	*n* = 14 adults	Digital patient portal for sharing with HCPs and third parties.	Patients expressed concerns about privacy and security risks. Patients were generally unaware of the meaning and value of health data to third parties which resulted in inconsistent views on data sharing. Patients desire granular control over their medical information but believe that this may negatively impact their quality of care. There is a need for more transparency about the potential consequences of sharing data with third parties.
Bussone et al. (18)	UK	This study investigated the TIPS considerations that people living with HIV make when sharing data with their peers for the purpose of guiding the development of trusted digital tools.	HIV	*n* = 26 adults	Digital health communities (sharing with peers)	TIPS concerns are central to those living with HIV when deciding whether or not to share personal health information with others. Platforms that are associated with a familiar HIV-related organization or charity benefit from enhanced trust. Robust privacy and security measures are key to ensuring trust in digital peer sharing platforms.
Caine and Hanania (38)	USA	The aim of this study was to assess patients' desire for granular level privacy; this includes control over which personal health information should be shared, with whom, and for what purpose. The study also addressed whether these preferences vary based on the sensitivity of health information.	Various conditions	*n* = 31 adults	Multiple recipients	Patients expressed a clear desire for control over which health information should be shared and with whom. Patients also expressed differences in sharing preferences for sensitive vs. less-sensitive health data.
Esmaeilzadeh et al. (39)	USA	This study aimed to examine the interplay between different chronic health problems and different types of sharing interfaces in relation to patient willingness to share personal health information with HCPs.	Chronic mental illness and chronic physical illness	*n* = 607 adults	Structure and unstructured interfaces for sharing personal health information with HCPs.	The results described how individuals managing physical illnesses and mental disorders both favor highly structured data entry interfaces for sharing personal data. Mental health patients perceived less psychological risk, and reported lower privacy concerns when using a well-structured data entry interface to record their PHI compared to an unstructured interface.
Fergie et al. (16)	UK	The aim of this qualitative study was to explore how engagement with user- generated content can support people with LTHCs, and to explore the factors that limit users' adoption of these technologies.	Diabetes (type unspecified) and Common Mental Health Disorders (CMHD)	*n* = 40 adults	Social media	This study highlighted the complexities of users' engagement with user-generated content for support in their experience of LTHCs. The findings highlight the range of considerations which influence production and consumption of health content *via* social media, particularly around identity management and integrating health content into everyday online practice.
Fuji et al. (29)	USA	The purpose of this qualitative study was to explore how patients with type 2 diabetes use an Electronic Health Record (EHR) to manage their information for the purpose of self-care.	Type 2 diabetes	*n* = 59 adults	HCPs *via* an EHR	Patients valued being able to store their medical data on one electronic record that was easily accessible. However, most participants did not share their data with HCPs. Patients expect HCPs to have full access to their data without having to personally disclose it. A strong patient-provider relationship is important for the effective adoption of EHRs.
Hartmann et al. (32)	Germany	The aim of this study was to investigate the self-monitoring and self-management of depression as well as to explore the data sharing preferences of potential users of digital platforms.	Depression	*n* = 668 adults	Mobile apps	Individuals with depression want to take control of sensitive data, they do not want to share with everyone - particularly third parties. Individuals are concerned about tracking, particularly when they perceive that being tracked to a specific place could be used against them.
Kelley et al. (33)	USA	The aim of this study was to investigate student perspectives on self-tracking of mental health and how personal data is used to support mental health and wellness management.	Mental health	focus group *n* = 14, survey*n* = 297 students (18–24 years)	Multiple recipients *via* self-tracking technologies	Students were motivated to share data with family and friends as a sense of ‘accomplishment' and sharing with peers was motivated by a sense of altruism. Tracking and sharing data with HCPs changed their experience of healthcare visits and improved communication and decision making.
Lafky and Horan (40)	USA	The aim of the study was to better understand the design implications for EHRs for people living with chronic conditions.	Various conditions	*n* = 28 adults	Electronic health record	Individuals are less concerned about the security of health data (compared with financial data). People living with disabilities are less willing to take measures to secure their health information.
Leventhal et al. (41)	USA	The aim of the study was to assess patient preferences for accessing PGData through a digital system, CareWeb.	Various conditions	*n* = 105 adults	HCPs	More than half of all participants wanted to share all of their data with HCPs. Only 5 participants out of 105 did not want anyone to view their data in the EHR.
Maiorana et al. (25)	USA	The aim of the study was to examine how trust (in tech, people and processes) influences the acceptability of data sharing in an HIV related context.	HIV	*n* = 549 adults	HCPs and other stakeholders *via* Health Information Technology (HIT)	People living with HIV are widely accepting of HIT. Increased experience and comfort with digital technology, confidence in security protocols, trust in providers and institutions who use the technology enhance understanding of the benefits to patients.
Murnane et al. (34)	USA	The aim of this study was to better understand how people living with Bipolar Disorder use data in condition management and how this may be facilitated by the use of personal informatics systems.	Mental health (Bipolar Disorder; BD)	*n* = 22 adults	Multiple recipients *via* self-tracking technologies	People with BD believe that sharing data with HCPs is standard and supports doctor-patient communication. Sharing with family and friends is important for recognizing when patients with BD may need intervention and support.
Nurgalieva et al. (42)	Sweden	This study explored patient perspectives on what technical, ethical, security, and privacy challenges need to be considered when designing platforms for sharing medical information.	Various conditions and a subgroup of cancer patients	Survey*n* = 2,587 adults Interviews of cancer patients *n* = 15 adults	A national online platform for accessing personal electronic health information and sharing with multiple recipients.	Few patients chose to share health information through an online platform despite a majority of patients trusting the security of the system. Cancer patients and psychiatric patients were notably hesitant to share online. Different conditions might cause a range of feelings in patients regarding sharing their health information, such as concerns about stigma.
O'Kane et al. (30)	UK	The purpose of the study was to explore how chronically ill patients and their specialized care network view their personal medical information privacy and how it impacts their perspectives of sharing their records with HCPs and third parties.	Diabetes (Types 1 and 2)	*n* = 27 adults	Multiple recipients *via* Health Information Technology	Diabetes patients shift their perceived privacy concerns and needs throughout their lifetime due to the persistence of health data, changes in health, digital technology advances, and experience with technology that affect one's consent decisions around privacy.
Teixeira et al. (26)	USA	The aim of this study was to assess the attitudes of individuals living with HIV/AIDS toward having their personal health information stored and shared electronically.	HIV	*n* = 93 adults	Health Information Technology (HIT)	The majority (84%) of individuals were willing to share their PHI with clinicians involved in their care. Fewer individuals (39%) were willing to share with non-clinical staff. Willingness to share PHI was positively associated with trust and respect for clinicians.
Torabi and Beznosov (43)	USA	This study explored perceptions of privacy risk when sharing personal health information *via* online social networking sites.	Various conditions	*n* = 166 adults	Social media	The results suggest that the majority (over 95%) of participants share some form of health or lifestyle information, with the “type” and the “recipient” of the shared data being the key factors that affect the perceived privacy risk and the risk-mitigating behavioral responses.
Vaala et al. (31)	USA	This study aimed to understand the willingness of adolescents to share type 1 diabetes (T1D) information with their peers.	Type 1 diabetes	*n* = 134 adolescents (12–17 years)	Sharing with peers *via* social media	Adolescents were more willing to share how they accomplished T1D tasks than how often they completed them, and least willing to share glucose control status. Sharing/helping beliefs and glucose control were related to greater willingness to share personal health information.
Warner et al. (44)	UK	This research looked at the app Grindr and the concerns around HIV disclosure for men living with HIV.	HIV	*n* = 149 adults	Grindr	The study finds some HIV positive users report keeping their status private to reduce their stigma exposure, whilst others report publicly disclosing their status to avoid being stigmatized by others. Where users keep their status private, concerns that social assumptions may develop around these non-disclosures, create a privacy unraveling effect which restricts disclosure choice.
Weitzman et al. (45)	USA	This study aimed to test the willingness of an online diabetes community to share data for public health research by providing members with a privacy-preserving social networking software application for rapid temporal geographic surveillance of glycaemic control.	Diabetes (type unspecified)	*n* = 1,136 adults	Health surveillance technology (mimicking social networking sites)	Users self-enrolled to use the digital technology and of those who enrolled, 83% added up-to-date glucose data. Sharing was high with 81.4% of users permitting data donation to the community display. 34.1% of users also displayed their glucose data on their profile page. Users selecting the most permissive sharing options had a lower average A1c (blood glucose level) (6.8%) than users not sharing with the community 95% of users permitted re-contact.
Zhang et al. (46)	China	This study looked at the sharing of personal health information in online health communities for people living with multiple conditions.	Various conditions	*n* = 337 adults	Sharing with peers *via* online health communities	Health information privacy concerns, together with informational and emotional support, significantly influence personal health information disclosure intention. Privacy concerns are negatively influenced by two coping appraisals (i.e., response efficacy and self-efficacy) and positively affected by two threat appraisals (i.e., perceived vulnerability and perceived severity).
Zhu et al. (47)	USA	This study looked at the use of patient-generated data using digital technology in a clinician-patient consultation.	Various conditions	*n* = 12 adult patients *n* = 9 clinicians interviews	Self-tracking technologies and sharing data with HCPs	Patients are motivated to collect and share PGData to foster a better understanding of their health and improve clinician appointments. Clinicians largely ignored data brought to consultations in this study. Some clinicians and patients feel overwhelmed by raw data.

From the selected studies, many focus exclusively on specific LTHCs: diabetes (types 1, 2 and unspecified; *n* = 4), HIV (*n* = 4) and mental health (*n* = 4). One study specifically addresses patients who manage multiple chronic conditions (MCC) and the remainder of the studies comprise participants who have a range of different LTHCs (*n* = 10). One study looking at type 1 diabetes reports the perspectives of adolescent participants (12–17 years) (31) and the remaining studies are of adults participants (18–84 years). The majority of the included studies explore the sharing of PGData with healthcare providers and electronic health record management (17, 26, 29, 30, 33, 34, 36, 40, 41, 47), with some including sharing of data with a wider network including public health and researchers (38, 45). Three of the studies look at the implications of sharing PGData online through social networking sites such as Facebook (16, 31, 45). One study looks at Grindr and the sharing of HIV status (44), whilst the other HIV related studies look at health information technology more broadly (25, 26). The following sections discuss the results in relation to the research questions driving the review.

## RQ1: Do Tips Concerns Present A Barrier to the Successful Sharing of PGDATA Using Digital Technology by People Living With Long-Term Health Conditions?

This narrative review finds that multiple TIPS concerns present barriers to the sharing of PGData *via* digital technology by those living with LTHCs. Distrust in the proposed recipient of PGData inhibits sharing *via* technology. Trust is often shaped by patients' previous experiences of sharing and, in a clinical context, can be facilitated by confidence in the healthcare institution or team with whom sharing is proposed. The desire by patients to control and self-manage their digital identity also impacts on patient willingness to share PGData with others. However, the review suggests that the use of pseudonyms can offer a successful strategy for facilitating sharing of PGData online by those living with LTHCs. Privacy and security concerns present clear barriers to sharing PGData *via* technology. Privacy concerns are reported as being the main reason patients may choose not to share PGData in a clinical context, though these concerns mostly relate to the potential for future sharing with external third parties. Anticipated security breaches by patients also present a barrier to the sharing of PGData with others, whereas believing that digital technology has sufficient safeguards in place is a facilitator to sharing PGData *via* technology. A more detailed discussion of individual TIPS concerns is given below.

### Trust

Here we address the degree of trust or distrust that is established between an individual and the proposed recipient of their PGData. A quarter of the articles discussed ‘trust' in relation to the sharing of PGData (17, 18, 26, 30, 31, 33, 42, 44). In the majority of these papers, trust as a barrier to the sharing of PGData centered on *distrust of the recipient*. When sharing with healthcare providers and clinical staff, distrust can be shaped by previous negative experiences for people living with multiple chronic conditions (17). Distrust is also developed when patients are asked to provide information that they deem to be highly personal and irrelevant to the given context (30). On the other hand, developing and building trust with recipients is considered a facilitator to the sharing of PGData and is supported by familiarity and confidence in the healthcare institution and healthcare team (25, 26). Where, for example, Teixeira et al. (26) report on willingness to share data for patients living with HIV:

“*Patients reported having a great deal of trust in their HIV care team. Trust in their care team to deliver high-quality medical care and feeling that providers spent enough time with them were each associated with patients' willingness to share PHI [protected health information] with both clinical and nonclinical staff at their primary clinic”* (26)

The majority of the papers examine PGData sharing within a clinical context, focusing on the barriers and/or facilitators to sharing with HCPs *via* digital technology. In this setting, trust is a key issue that makes patients more likely to share PGData with trusted recipients. Kelley et al. (33) report how sharing PGData improved the relationship and trust between patients and their clinicians, with student participants reporting how they used PGData to provide proof that they were doing exactly what they said they were. We know that higher levels of patient trust in HCPs are associated with more beneficial health behaviors, fewer symptoms, and higher quality of life (48). Conversely, a lack of trust in HCPs can prevent patients from sharing some forms of PGData and engaging with HCPs in face-to-face settings (49).

This review indicates that trust remains an important factor in PGData sharing *via* digital technology. Most papers focussing on a clinical setting examine data provided by patients that constitutes personal health information that they have chosen to incorporate into their electronic health records (EHR). In general, these studies indicate patients are happy to share most information with HCPs but less so with non-clinical staff (26). The focus on the EHR as a digital artifact provides common ground for the patient and the HCP. Shared data can underpin improved communication between patients and HCPs encouraging a more patient-centered approach although such artifacts also have the potential to disrupt the doctor-patient relationship (50). The few papers that focus more on the sharing of self-tracking data with clinicians (33, 47) contrast the perceived benefits experienced by patients with the more negative or skeptical feelings toward the data expressed by HCPs.

Trust as a barrier to sharing is discussed less often outside of the context of sharing with HCPs. A notable exception is Warner et al. (44). In discussing the importance of mutual self-disclosures in the development of trust, Warner et al. (44) note that the features of mobile apps do not always support trust in their users. Uncertainties over the disclosure of patient-provided health information (i.e., HIV status in the mobile app dating environment, whereby people do not disclose, or report their last sexual health check as a long time ago) can cause distrust of other people living with HIV. A further study which addresses the role of trust outside of a clinical context is provided by Bussone et al. (18). This study explores the concerns of those living with HIV when sharing personal health information with their peers and finds that trust in digital sharing platforms can be enhanced when it is associated with a recognized HIV charity or trusted medical organization. This study also describes how strong privacy and security measures are vital for building trust in such peer-sharing platforms.

### Identity

The literature discusses digital identity in terms of concerns regarding identifiers relevant to one's personal data and online presence. The conscious management of digital identity online has an impact on patient willingness to share PGData with online social networking sites such as Facebook (16, 30, 31, 43, 44). People living with diabetes, mental health or HIV expressed a desire to withhold PGData relating to their condition from their wider social network (16, 31, 44):

“*Many participants reflected on the undesirability of contributing any health-related content to Facebook, since this platform was seen primarily as a space for the conscious construction of a positive identity. As such, the inclusion of references to diabetes or mental health could jeopardise this*.” (16)

This is further supported by Bussone et al. (18) who explore attitudes toward sharing among those living with HIV and find that participants report a strong desire to self-manage certain aspects of their digital identities by sharing individual attributes of identity if anonymised:

“*They indicated willingness to share digital identity attributes, including gender, age, medical history, health and well-being data, but not details that could reveal their personal identity*.” (18)

An alternative strategy for managing digital identity is discussed by O'Kane et al. (30) who describe how some people living with either type 1 or type 2 diabetes are happy to share their PGData under pseudonyms in specific health related online forums provided they get the support they need in return:

“*The use of social media seems to be a fine balance between openly sharing sensitive medical information whilst also remaining in control of what is considered private. If you want to talk about the worst thing that you've done to your diabetes, or you are really ignoring it, or you're in a dark place, you can share that information without sharing your name, without alerting your employer to your potential issue or alerting your family even. You can keep those feeling private but share them publicly in a way gets the support without putting you out there like you're waving a flag saying ‘I'm diabetic and I want everyone to look at me!' right? – Patient 14*” (30)

The management of digital identity is closely linked to how well patients manage their condition, even when seeking out support. When the perceived management of the condition is considered poor, some patients are less likely to share their data. Among adolescents with type 1 diabetes, Vaala et al. (31) report, “*Those who consider posting health-related information online face a tension between pursuing health-related goals, such as obtaining advice or emotional support, and maintaining a favorable impression as someone who is healthy and competent it seems the balance may shift in favor of the latter among adolescents who are struggling with glycemic control*.” Other studies investigating the sharing behaviors of people living with diabetes (type unspecified) with public health researchers have discovered that patients with better self-reported measures of glycaemic control are more likely to share their data (45).

Warner et al. (44) report on the reflection of HIV disclosure and identity management as some study participants note how they perceive the sharing of a person's negative HIV status and last test date as a way to show off to other users on Grindr, where one participant states, “*I just don't like it. It's like giving yourself a pat on the back for being lucky or” “better” “than other people”*.

In terms of sharing PGData with online social networking sites, identity and privacy are key issues. People living with LTHCs want to be able to withhold PGData relating to the condition from their wider social network and to exert control over what data they share and with whom. For people with LTHCs these needs reflect changing patterns of engagement with social networking sites and online support groups (51, 52). Sharing PGData may occur in a temporary or intermittent manner, depending on the nature of condition and the type of PGData shared, which often varies in relation to the stage of the illness or health condition (53). Many people with LTHCs are less likely to share PGData when they are perceived to be managing their condition poorly (45) and blaming and shaming can often be a core experience for people with diabetes on online forums (54).

“Digital personhood” (a term used to discuss recognition of a human being as having status as a person in the electronic realm) can be impacted by illness, resulting in pre-and post-illness personas (55). Managing our identities across different contexts is often difficult when engaging in social interaction online, a term recognized as “context collapse” (56). People with LTHCs may have to work harder at their online communication, making more conscious decisions about what PGData to share and what to withhold, in order to shape or maintain their preferred digital identity or presentation of self (57). Separating out more generic social networking sites such as Facebook from specific, often anonymous, online health support groups is one strategy. Newman et al. (58) show how people with LTHCs manage their PGData sharing between online health communities and Facebook; Facebook is used to present a positive identity of self-control, whilst an online forum, by contrast, affords a space to be more open about expressing personal difficulties.

### Privacy and Security

Privacy and security issues refer to concerns raised by patients surrounding the preservation of individual privacy and the ability to provide secure storage of personal data and information. Privacy concerns are discussed as a barrier to the sharing of PGData in the majority of articles. Agaku et al. (36) report that privacy and security concerns are the main reason why some patients withhold their PGData from healthcare professionals. In addition, the authors report concerns about the security of information whilst being “electronically transferred” or ‘faxed', as well as ‘the perception that a patient had very little say in how their PGData was used' are all associated with significantly higher odds of withholding personal information from a healthcare professional (36). Similarly, Caine and Hanania (38) report that patients express having less choice over what is shared with third-party organizations, e.g., health insurance companies. The request by patients for granular control over sharing of PGData and medical information is common across many articles (29, 30, 36–38, 44) and informed consent is requested to enable the patient to make decisions about who to share their data with (36, 38). Bernaerdt et al. (37) find that this desire for granular control in certain patient groups is often present despite a lack of awareness of the value or meaning of medical data to third parties. This evidence suggests that patients need to be better informed of the consequences and implications of sharing personal health information with third parties.

Torabi and Beznosov (43) note that privacy risk perceptions of people living with LTHCs are context dependent. Many authors also highlight the perceived sensitivity of PGData to the patient, and that how a person feels about their physical and mental health at the time of sharing impacts privacy risk perception (30, 32, 38, 45, 46). One particular study looking at multiple conditions and sharing PGData from Electronic Health Records (EMR) reports,

“*There was not one potential recipient (e.g., primary care physician) with whom all patients wanted to share all of the information in their EMR with unconditionally. This was the case for both groups of participants: those with highly-sensitive health information in their EMR (21 participants) and those without highly-sensitive information (nine participants)*.” (38)

However, some patients expect healthcare professionals to have complete access, despite the sensitivity of data, “*they need to know everything that is going on in your health*” (30).

Hartmann et al. (32) describe how patients may wish to minimize the potential risk of data being used against them by third-party organizations:

“*Individuals want to keep control of such sensitive data and just do not want to share it with everybody or more precisely with third-party agents from whom negative consequences could arise from, such as German public health insurance, for instance. People are worried about being tracked at places that indicate risk behavior or self-damaging behavior, which could result in financial consequences (e.g., higher insurance rates or loss of treatment reimbursement)*.” (32)

Concerns over sharing PGData with HCPs typically focus on the potential for the data to be shared more widely with third-party organizations, and the review indicates that patients are keen to be able to control or limit this wider sharing to protect the privacy of their data.

On social media use for diabetes support, O'Kane et al. (30) report patients' changing perspectives on privacy, where social media use is a delicate balance of sharing openly sensitive medical information whilst also having control over what is considered private, based on how vulnerable they feel:

“*People may choose to view previously held privacy beliefs as overly cautious and want to reveal more about their previous medical history, but they still have their own individual levels of comfort. Although Patient 13 would write his diabetes blog under his own name and picture as mentioned above, one group interview participant did not feel comfortable with this level of privacy. I think it would be alright to share information about how your, maybe how your blood sugars go… […]but I don't think it is necessary to say your name and your address or anything like that. You can have a blog where everyone has a username or something. And then I think it's really helpful. I don't think you really need to identify yourself. – Group Interview Participant*” (30)

However, sometimes the interest in maintaining dignity and privacy (on any digital platform) can outweigh the interest in health and subsequently results in patients withholding PGData (30).

Privacy and security concerns are shown to be significantly influenced by particular demographics (e.g., age and education level), and characteristics (e.g., self-efficacy) (46), as well as the trajectory of a person's illness and “*other temporally-situated outside influences*” (30). Furthermore, differences between LTHCs may influence the extent to which privacy concerns influence sharing preferences and behaviors. For example, Esmaeilzadeh et al. (39) describe how differences between mental and physical conditions result in differences in sharing propensities:

“*Individuals with a physical illness favor higher levels of structure mainly due to information quality dimensions (i.e., better understandability, accessibility, and usefulness). However, individuals with mental disorders prefer highly structured interfaces due to lower psychological risks and privacy concerns*.” (39)

Nurgalieva et al. (42) also highlight how different conditions may elicit a range of privacy concerns. They show how cancer patients and psychiatric patients were notably hesitant to share *via* a national digital platform for the sharing of personal health information. This may be explained by certain conditions being more likely to provoke fears surrounding potential stigma or causing family members to worry (42). Further understanding of the influence of both demographic and health condition factors is required so that healthcare organizations may adequately structure their patient platforms to accommodate the differing privacy concerns of patient groups, for example by providing information to patients about how data is going to be used and stored.

Anticipated security breaches present a barrier to the sharing of PGData (30, 36), whilst in contrast, having confidence that digital technology has safeguards in place is a facilitator to sharing of PGData (36). Patients' concerns are justified by factors including their previous experiences of digital technology and security breaches occurring both electronically and using paper health records (30).

Privacy concerns affect sharing PGData in online settings. People with LTHCs have to make judgements about the type and amount of information they share with others, weighing up the contextual integrity of their personal data sharing against potential privacy and security posed by the “silent listeners” on the network, i.e., third-party applications or advertisements (59). Site ownership and funding plays into this directly with peer-sharing resources now being hosted by large pharmaceutical companies, charities, healthcare organizations and individuals. Some data-driven sites such as PatientsLikeMe have been built to support information exchange between patients (11) but their relationship with third-party organizations can cause some users to feel uncomfortable (60). Recent changes to the ownership of such sites may increase concern in this context; for example the acquisition of PatientsLikeMe by the healthcare and insurance company UnitedHealth Group caused some users to express privacy and security concerns regarding their personal data (61).

In comparison to sharing with HCPs or sharing *via* social media, there are relatively few papers that focus on sharing PGData within a third-party context. The papers that do examine this context identify privacy and security as key issues (30, 32, 37) and highlight that some patients may have little understanding of the value of PGData to third parties (37). However, clearly more work is needed to understand whether the TIPS barriers and facilitators play a role within this setting. The key messages in this setting are that people want to be able control the privacy of their data and to have the option of changing their consent preferences with regard to sharing. Patients are also more likely to share with organizations that have the potential to impact their health directly and less likely with organizations further from this premise (i.e., researchers, government or health insurance companies). Although the papers examine patients' attitudes toward sharing PGData with third-party organizations, they do not explore differences in sharing behaviors depending on whether or not PGData is anonymised.

## RQ2: What is the Impact of Stigma on the Sharing of PGDATA *Via* Digital Technology by Those Living With LTHCS?

Stigma can be both internal (felt stigma or self-stigmatization) or enacted (external or discrimination) experiencing unfair treatment from others (62). Anticipated stigma presents a barrier to the sharing of PGData, across multiple platforms and with various recipients (18, 30, 31, 36, 44). A range of health conditions are associated with significant stigma (63), such as living with HIV (18, 64), mental health problems (65, 66), and chronic pain (67). People living with LTHCs are at risk of losing out on the benefits of sharing data when affected by stigma and are more likely to withhold information. Both internal and enacted stigma impact the way in which patients develop trust with the recipients of PGData.

Internal and enacted stigma can create a barrier to sharing PGData, particularly for people living with HIV. When exploring the use of Grindr to disclose HIV status, Warner et al., (44) report how people living with HIV are sometimes keen to withhold this information due to concerns of social exclusion and loss of sexual opportunity. Although in contrast, the article also describes some comments from Grindr users about how stigma can be used as a motivator for disclosure for some men living with HIV as a way to “reduce their stigma exposure”. However, Warner notes,

“*Stigma around HIV could lead some users to purposefully misreport their HIV status to avoid exposure to stigma. This is reflected in our findings, where users report their desire for HIV disclosure choice. In an environment where all users are expected to disclose, privacy unravelling around non-disclosures may limit this choice. When all said and done, it's forced disclosure that I dislike, or the fact that HIV*+ *users are expected to self-disclose their status straight away. Why should they? (Paraphrased comment from NW8)*.” (44)

The majority of findings relating to stigma are of people living with HIV (18, 25, 26, 44). However, in other conditions, authors note how participants express their concerns over their PGData being used against them by healthcare providers and third-party organizations:

“…*A woman with a previous psychiatric diagnosis believed her history had been misused by ambulance personnel who “put my name in the computer” and diverted her to psychiatric care instead of the medical emergency care she was seeking. Another individual was concerned about how doctors interpreted the history of sexually transmitted infection in his medical record. One woman was strongly motivated to conceal her diabetes from her insurer because she was concerned the company would raise her premiums*.” (17)

“*Individuals want to keep control of such sensitive data and just do not want to share it with everybody or more precisely with third-party agents from whom negative consequences could arise from, such as German public health insurance, for instance. People are worried about being tracked at places that indicate risk behavior or self-damaging behavior, which could result in financial consequences (eg, higher insurance rates or loss of treatment reimbursement)*.” (32)

Among adolescents with type 1 diabetes, an increase in restrictive sharing settings through social media are considered a factor of anticipated stigma when adolescents have higher than normal blood glucose levels (31, 45). Insights into the sharing preferences of previously explored groups, such as those living with HIV and diabetes, may help to guide the further study of the role that stigma plays in the formation of attitudes and sharing behaviors in those living with other LTHCs.

## Discussion

### Summary of Findings

Trust, Identity, Privacy and Security (TIPS) concerns can present a barrier to sharing health and lifestyle data when using digital technology to share data in multiple contexts. A quarter of the articles discussed the role of trust in sharing PGData. Privacy as a barrier to sharing was present across most articles and across most settings. Other TIPS concerns were more readily identified as barriers to sharing in certain contexts. Identity management was seen as a barrier to sharing more frequently within the context of social networking sites and the issue of security was a barrier to the sharing of PGData with third parties. The presence of enacted stigma acted as a barrier to sharing PGData across all settings although this was most noticeable in relation to HIV compared to other LTHCs.

The narrative review has shown that TIPS issues are a considerable barrier to the sharing of PGData across all settings. The presence of specific TIPS issues varied by context, such that in certain settings particular barriers were more prominent. However, the literature shows that the majority of research looking at the sharing of PGData has focused on clinical settings with relatively few studies examining attitudes toward sharing with third parties such as public health and research. In clinical settings the key TIPS issue was trust. Distrust in the recipient of the information was highlighted as a key barrier to sharing PGData *via* digital technology.

In social network sharing online, we found that identity and privacy concerns were expressed in relation to the self-management of health and concerns regarding oversharing. These issues were key barriers to sharing but there was a lack of more detailed and nuanced information about the kind of PGData individuals were or were not sharing with respect to these concerns. Whilst the focus of this review paper was on the barriers and facilitators of sharing PGData more broadly rather than types of data *per se*, it was interesting to note that the studies covered a range of PGData. In clinical settings, unsurprisingly the focus was on electronic health records and clinical data, whereas in the social networking settings, the range of PGData was more varied and included more subjective data around mood, sleep and emotions. Despite focussing on stigmatized health conditions, there was relatively little focus on the role stigma played in decisions regarding sharing PGData *via* digital technology. References to stigma were most prevalent in relation to HIV but far less mentioned with respect to other conditions. Understanding the roles of both internal and enacted stigma regarding the sharing of PGData needs further attention. Much of the discussion surrounding stigma related to the unwanted disclosure of sensitive information. Despite a lack of consensus about what should be considered sensitive information, previous literature suggests five categories of sensitive health data: sexually transmitted infections, HIV/AIDS status, sexual health and pregnancy, mental health information, and substance use (41). However, legal definitions of what constitutes sensitive personal data are often very broad in scope; for example, the European Commission categorizes “health-related data” as sensitive personal data (68). Further research may seek to examine how perceptions of information sensitivity among those with various LTHCs affect patient privacy concerns and explore how these concerns may vary across different conditions.

Whilst we have assumed that sharing is a beneficial activity, it is also worth considering that, as part of supporting the management of PGData, we need to think about how people make sense of their data. We cannot always expect people to be able to successfully interpret their data (34), and collecting and monitoring data can be overwhelming for some people leading to negative health consequences (69). Patients may express varying preferences for managing PGData and have different technological abilities relevant to the skills required to actively record, monitor and manage personal health information. Understanding these patient differences may help to avoid burdening people with the “invisible work” of managing personal health information (17, 70). Managing PGData can also add to the increasing demands faced by HCPs due to the time required to analyse and make sense of the data that patients provide. As well as understanding the role of health literacy in relation to managing PGData (17), and the burden placed on both patients and HCPs, we need to know more about the motivations for both collecting and sharing PGData in different contexts to see if TIPS issues vary accordingly. Understanding more about the types of PGData people with LTHCs are happy to share and how the TIPS barriers might differentially apply to these forms of data would be a useful next step. Finally, there is a need for more qualitative studies in this area, especially in relation to TIPS barriers and facilitators to sharing PGData with third-party organizations as the majority of these studies are based on quantitative data.

Whilst our review highlights some of the key TIPS concerns that people living with LTHCs have with respect to sharing their PGData, none of the studies evaluated solutions or interventions to overcome these barriers. A few papers discussed participants' suggestions or desires concerning greater transparency and control over the information. Clearer informed consent to improve the transparency of the sharing process would increase the granular control for participants (30). A growing body of literature, that is beyond the scope of this narrative review, continues to explore technology and policy-based solutions to resolve general concerns about health data to facilitate secure and privacy-preserving sharing (71–73). However, given the specific TIPS concerns that this narrative review highlights with respect to the sharing of PGData by those living with LTHCs, future research may look to investigate how successful those solutions proposed to tackle general concerns about health data are at alleviating the TIPS concerns of those living with LTHCs. Furthermore, though recent research examining dynamic consent models for the sharing of clinical data (blood and tissue samples) in third-party contexts showed promising results in terms of acceptability (74), it remains to be seen how such models would work across more stigmatized health conditions and across more varied PGData types. Although there is still little empirical work in this area, the UK EPSRC funded programme INTUIT is examining TIPS concerns around PGData sharing primarily for people living with HIV but also for those with other stigmatized conditions. The INTUIT project aims to identify TIPS concerns and to design tools that remove the barriers to collecting and sharing PGData in order to improve the health and well-being of stigmatized populations. As part of this project, we are conducting interviews with people living with LTHCs to examine the role of sharing context and health condition in relation to TIPS barriers. This is the first study of its kind to focus specifically on TIPS issues in relation to sharing PGData *via* digital technology across a variety of stigmatized LTHCs and across a range of different sharing contexts.

## Conclusion

This narrative review has provided a broader perspective on the TIPS challenges faced by people managing LTHCs and has shown that TIPS issues are a considerable barrier to the sharing of PGData *via* technology by those living with LTHCs across all settings (i.e., sharing with clinical staff, public health surveillance, researchers, peer communities, friends, social networks and other third-party organizations). Distrust in the proposed recipient of PGData, the need to manage one's digital identity and broadly held privacy and security concerns present barriers to sharing in a clinical setting but more research is needed to understand other contexts, particularly sharing with third parties. The presence of internal and enacted stigma has also been shown to impede the sharing of PGData across all settings, although most research in this area has centered on those living with HIV. This highlights the need for further research to consider differences between conditions in experiences of stigma, and to consider how these differences interact with the influence that TIPS concerns have over sharing. Whilst the technological sharing of PGData holds great potential benefits for the health, well-being and social outcomes of people managing LTHCs, the TIPS challenges faced by those individuals must be better understood and addressed if interactions with care services, peer support networks, and private organizations are to be optimized.

## Data Availability Statement

This paper provides a narrative review of existing literature. All data underlying this review are cited in the references.

## Author Contributions

The initial concept for the project was founded by LC, ES, and AD. The research questions and search criteria were then developed by LC, ES, and EmS and reviewed by all authors. EmS conducted the initial search and review and RB conducted the final search and review. LC and ES supported EmS in the shortlisting of papers against the criteria. EmS, RB, ES, LC, KL, JG, ST, and AD participated in discussing, revising and editing the manuscript.

## Funding

This work was funded by the EPSRC, grant number EP/R 033900/1.

## Conflict of Interest

The authors declare that the research was conducted in the absence of any commercial or financial relationships that could be construed as a potential conflict of interest.

## Publisher's Note

All claims expressed in this article are solely those of the authors and do not necessarily represent those of their affiliated organizations, or those of the publisher, the editors and the reviewers. Any product that may be evaluated in this article, or claim that may be made by its manufacturer, is not guaranteed or endorsed by the publisher.
